# Photosynthetic carbon from algal symbionts peaks during the latter stages of embryonic development in the salamander *Ambystoma maculatum*

**DOI:** 10.1186/1756-0500-7-764

**Published:** 2014-10-28

**Authors:** Erin R Graham, Zaid M McKie-Krisberg, Robert W Sanders

**Affiliations:** Department of Biology, Temple University, 1900 N. 12th St, Philadelphia, PA 19122 USA

**Keywords:** Algae, Symbiosis, Salamander, Carbon translocation

## Abstract

**Background:**

It was recently discovered that symbiotic algae in the eggs of the salamander *Ambystoma maculatum* translocate fixed carbon from photosynthesis to developing embryos. Fixed carbon translocation was shown in embryos at one time point during development, however, it was unknown if fixed carbon translocation occurs throughout all developmental stages.

**Findings:**

In this study, fixed carbon translocation was measured in salamander eggs at six time points over the latter half of development. Fixed carbon translocation did not occur until the middle tailbud portion of development (stages 26–30), and translocation was measured in 20% or less of eggs sampled. Peak carbon translocation occurred during the late tailbud phase of development (stages 31–35), where as much as 87% of eggs sampled showed translocation, and average percent translocation was 6.5%. During the final stages of development, fixed carbon translocation declined, and translocation was not detected in embryos five days prior to hatching.

**Conclusions:**

The onset of fixed carbon translocation from *Oophila* to *A. maculatum* embryos during the second half of embryonic development is likely due to the corresponding settlement and concentration of *Oophila* in the inner egg envelope. In addition, carbon translocation ceases in late stage embryos as the inner egg envelope thins and ruptures in preparation for hatching.

## Findings

### Background

The green alga *Oophila amblystomatis* (Lambert ex Printz) [1], inhabits the eggs of the North American spotted salamander, *Ambystoma maculatum. Oophila* releases photosynthetic oxygen in the egg capsules [2, 3] which helps reduce anoxia in the thick gelatinous egg masses. Increased oxygen in eggs accelerates embryonic growth and development, promotes synchronous hatching, and reduces embryo mortality [4–6]. *Oophila* also fixes CO_2_ though photosynthesis, and translocates fixed carbon to the developing salamander embryos [7].

Embryonic development is a continuous process, but in the genus *Ambystoma* it is characterized by forty-four to forty-six stages between fertilization and resorption of the yolk that can be defined by distinct morphological features (see [8, 9]). Although *Oophila* migrate into eggs only a few hours after the eggs are deposited, algal population densities increase significantly during the first 25 stages of development. During the latter stages of development, algae become non-motile and concentrated in the inner egg envelope adjacent to the developing embryo [7].

The recent discovery that photosynthetically-fixed carbon is translocated to *A. maculatum* embryos from *Oophila* symbionts has raised several questions. In this study, we further describe carbon flux in this symbiosis by identifying the onset of fixed carbon translocation, and measuring changes in the percentage of fixed carbon translocated throughout the second half of *A. maculatum* development.

### Methods

*A. maculatum* egg masses were collected on 23 March 2013 from Bucks County, PA, USA. Egg masses were transferred to the laboratory, rinsed, and maintained in an incubator at 8°C in fresh, dechlorinated tap water on a 12:12 light:dark cycle (~1.5 × 10^15^ quantas^-1^ cm^-2^). Embryos were staged using the staging series of Bordzilovskaya et al. [8] to enable direct comparison with Graham et al. [7]. The staging series of Bordzilovskaya et al. [8] is comparable to the staging series of R.G. Harrison [9]. The Temple University Institutional Animal Care and Use Committee (IACUC) does not require approval to work with larval (non-primate) vertebrates, therefore, this study conforms to institutional animal ethics regulations.

Carbon translocation from *Oophila* to *A. maculatum* embryos was measured as described in Graham et al. [7]. Briefly, for each of six experimental time points, eggs (n = 15) were incubated in 5ml dechlorinated water containing 3ml of sodium [^14^C] bicarbonate (final specific activity of 111 kBq per sample) in replicate scintillation vials. Vials were placed in the light (1.5 × 10^15^quantas^-1^cm^-2^) at 8°C. For each experimental time point, control samples of eggs (n = 15) containing embryos at the same developmental stage were incubated in the dark at 8°C. After 1.75 hours, eggs were removed, rinsed, and transferred to vials containing clean dechlorinated water. All vials (both light and dark incubated) were placed in the dark at 8°C for a two-hour dark phase period. We also repeated this procedure on stage 36 embryos that were carefully removed from their eggs and rinsed thoroughly to remove any surface algae before being incubated in radiolabeled water in the light and dark. After incubation, embryos contained in eggs were separated from their eggs by rupturing the egg (preserving all of the egg membranes and fluid) and removing the embryo using forceps. Embryos removed from their eggs were rinsed thoroughly, and homogenized in clean dechlorinated water. Subsamples of homogenized embryo only, and homogenized embryo plus egg capsule were acidified with 6mol l^–1^ HCl in a fume hood overnight to remove unincorporated ^14^C, then neutralized with 6mol l^–1^ NaOH. Scintillation fluid was added and samples were measured in a Beckman LS-3801 scintillation counter. Mean disintegrations per minute (dpm) of dark controls (embryo only and embryo plus total egg) were subtracted from their corresponding samples incubated in light. Percent translocation was calculated as (DPM embryo light- DPM embryo dark)/(DPM total egg light – DPM total egg dark), where the total egg fraction includes a homogenate of all egg membranes, algae, and the embryo.

Mean percent fixed carbon translocation was calculated for all samples (including those with zero translocation) at six developmental stages. In addition, the percentage of eggs/samples in which translocation was measured out of the total number of eggs/samples was recorded. Carbon translocation percentages were transformed to meet the assumptions of normality and equal variance by calculating the arcsine of the square root. Transformed percentage values were compared using oneway ANOVA with Tukey post-hoc analysis using JMP 10.0.0 statistical software (SAS, Cary, NC, USA).

### Results

**Figure 1:**
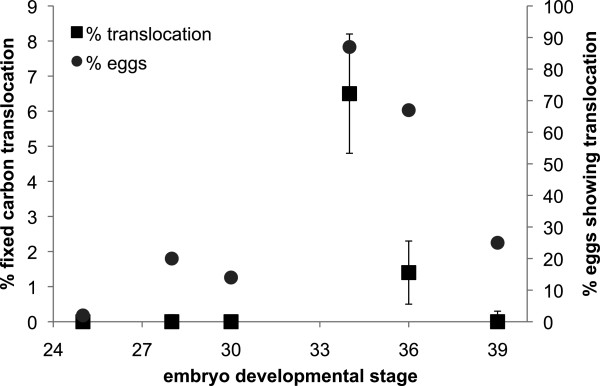
**Translocation of fixed carbon from** ***Oophila*** **to** ***A. maculatum*** **embryos during the second half of embryonic development.** Points represent means (*n* = 8-15 samples for each point). Error bars represent standard error (shown for % fixed carbon translocated only).

Fixed carbon translocation did not occur in any eggs in early tailbud embryos (developmental stage 25) (Figure 1). In middle tailbud embryos (stages 28 and 30), fixed carbon translocation only occurred in 20% and 14% of eggs sampled, respectively (*n* = 15 for each), and mean percent translocation of carbon was not significantly different than zero (*p* < 0.0001). Fixed carbon translocation did not occur in the majority of the eggs sampled until embryos were in late tailbud phase (stage 34), at which time, 87% of eggs sampled had measureable carbon translocation (*n* = 15). An average of 6.5% (+/- 1.7% S.E.M.) of carbon fixed by *Oophila* was translocated to stage 34 embryos (Figure 1). Fixed carbon translocation was also measured in 67% of stage 36 embryos, however, the amount translocated decreased to 1.4% (+/- 0.9% S.E.M.), which was significantly lower than translocation at stage 34 (*p* = 0.0265). Finally, only 25% of embryos had measurable translocation at prehatching stage 39, which resulted in mean translocation not significantly different than zero. Mean translocation for all eggs that had measureable translocation across stages (28, 30, 34, 36, 39; *n* = 25), was 6.5% (+/- 1.4% S.E.M.). For stage 36 embryos removed from their eggs prior to incubation, DPM was not different between embryos in the light versus those incubated in the dark (mean DPM 6141.6 +/- 979.9 S.E.M. for light and 6037.7 +/- 790.4 S.E.M. for dark).

### Discussion

The discovery that green algal mutualists translocate photosynthetically-fixed carbon to salamander embryos marked the first report of carbon translocation from a photosynthetic symbiont to a vertebrate host [7]. This raised additional questions about the nature of this energy transfer. In the current report, we addressed two of these major questions: 1) does carbon translocation occur throughout all stages of *A. maculatum* development, and 2) does the percentage of fixed carbon translocated change throughout development? Our results show that carbon translocation does not occur during the first half of embryonic development (stages 1–23). During the second half of development (stages 24–44) fixed carbon translocation is detected in a small fraction of embryos during the middle tailbud phase (stage 25), and the percentage of embryos receiving fixed carbon increases from stage 25 to stage 34. Second, the percent of fixed carbon translocated (including eggs in which no translocation was measured), varied among developmental stages. The mean peak translocation percentage was 6.5% (+/- 1.7% S.E.M.) and occurred in stage 34 emrbyos. This value is very close to the mean translocation value reported previously (6.4% +/- 1.3% S.E.M.) for stage 32–36 embryos [7].

There are several possible reasons why fixed carbon translocation does not occur until the latter half of embryonic development in the *Oophila*-*A. maculatum* association. First, from fertilization to early tailbud phase (ending at stage 25), most *Oophila* are motile and are located in the outer and middle egg membranes. *Oophila* are not in direct contact with the embryo at this point, therefore, carbon fixed during photosynthesis would not be easily transferred to the embryo. Even if *Oophila* released photosynthate into the egg capsule, it would need to pass through the inner egg membrane to reach the embryo. Moreover, higher energetic demands associated with motility may prevent *Oophila* from “giving up” any fixed carbon during the first half of embryonic development. Following stage 25, *Oophila* lose motility and concentrate in the inner egg envelope directly surrounding the embryo, or even on the surface of the embryo [7, 10] which could facilitate translocation from *Oophila* to the embryo. Changes in *Oophila* population density could also account for differences in translocation percentages from early development to late development. As shown in Graham et al. [7], *Oophila* population density increases significantly in eggs from stage 25 to stage 34. This higher abundance may make fixed carbon translocation easier to detect. However, Graham et al. [7] found no significant difference in *Oophila* population density among embryos at stages 34, 40 and 42.5 despite a reduction in translocation after stage 34 seen in this study. Peak translocation at stage 34 may be primarily a result of algal migration to the inner egg envelope and loss of motility (settlement), aided by an increase in *Oophila* population density.

As shown in this study, fixed carbon translocation decreases during the final stages of development. This is not surprising given that during the prehatched phase (stages 36–43), embryonic twitching and movement occurs as embryos prepare to hatch and swim out of their egg mass. During this final phase, egg membranes become more delicate and thin to facilitate the exit. Embryonic movement often causes the inner envelope and vitelline membrane to rupture several days prior to hatching (Graham, personal observation). Loss of direct contact with *Oophila* when the inner envelope is ruptured and *Oophila* are released back into the middle and outer egg membranes may prevent fixed carbon translocation (passive or active).

Changes in the number of eggs/embryos in which fixed carbon translocation is detected is likely due to variation in developmental stages and *Oophila* abundance among eggs and egg masses. In this study, eggs were selected from ten different egg masses that were at the same developmental stage. It is possible, however, that slight variation in developmental stage among embryos in the same egg mass may have resulted in some embryos being one or two stages earlier or later than reported. Thus, a few embryos within a mass may have been slightly more developed, and translocation may have begun in these eggs. Also, *Oophila* population density varies among eggs and egg masses at the same stage of development [7], which can affect carbon translocation. Finally, the time at which *Oophila* reach the inner egg envelope and lose motility may vary slightly among eggs at the same developmental stage.

This study provids additional evidence that fixed carbon from photosynthesis is translocated to *A. maculatum* from algal symbionts in the egg capsule. First, embryos in eggs incubated in ^14^C in the light had higher activity than embryos in eggs incubated in ^14^C in the dark. Second, embryos removed from their eggs prior to incubation in ^14^C in the light and in the dark had similar background levels of radioactivity whether they were incubated in the light or in the dark. This supports the hypothesis that the source of fixed carbon measured in *A. maculatum* embryos is not intracellular *Oophila*, or algae that have penetrated the embryo, but is capsular algae. Finally, radioactivity in embryos removed from their eggs prior to incubation was significantly lower than radioactivity in embryos incubated in their eggs (and for which translocation was measured). These findings, in addition to previous evidence [7] demonstrate that fixed carbon from capsular *Oophila* is entering *A. maculatum* embryos. However, the exact mechanism by which this translocation occurs remains unknown.
